# Associations of Polymorphisms in *MTHFR* Gene with the Risk of Age-Related Cataract in Chinese Han Population: A Genotype-Phenotype Analysis

**DOI:** 10.1371/journal.pone.0145581

**Published:** 2015-12-21

**Authors:** Xue-bin Wang, Chen Qiao, Li Wei, Ya-di Han, Ning-hua Cui, Zhu-liang Huang, Zu-hua Li, Fang Zheng, Ming Yan

**Affiliations:** 1 Center for Gene Diagnosis, Zhongnan Hospital of Wuhan University, Wuhan, Hubei, China; 2 Department of Ophthalmology, Zhongnan Hospital of Wuhan university, Wuhan, Hubei, China; 3 Department of Clinical Laboratory, Children's Hospital of Zhengzhou, Zhengzhou, Henan, China; Renal Division, Peking University First Hospital, CHINA

## Abstract

Homocysteine (Hcy) is a potential risk factor for age-related cataract (ARC). Methylenetetrahydrofolate reductase (MTHFR) is the key enzyme for Hcy metabolism, and variants of *MTHFR* may affect MTHFR enzyme activity. This study mainly evaluated the associations between variants in *MTHFR* gene, plasma MTHFR enzyme activity, total Hcy (tHcy) levels and ARC risk in Chinese population. Four single nucleotide polymorphisms (SNPs) in *MTHFR* gene were genotyped using the high-resolution melting (HRM) method in 502 ARC patients (mean age, 70.2 [SD, 9.0], 46.0% male) and 890 healthy controls (mean age, 67.1 [SD, 11.1], 47.6% male). The plasma MTHFR activity, folic acid (FA), vitamins B12 and B6 levels were detected by enzyme-linked immunosorbent assays (ELISA). The plasma tHcy levels were measured by an automated enzymatic assay. After the Bonferroni correction, the minor allele T of SNP rs1801133 showed a significant association with an increased risk of overall ARC (OR = 1.26, P = 0.003). Consistent association was also found between SNP rs1801133 and cortical ARC risk (OR = 1.44, P = 0.003). Haplotype analyses revealed an adverse effect of the haplotype "C-A-T-C" (alleles in order of SNPs rs3737967, rs1801131, rs1801133 and rs9651118) on ARC risk (OR = 1.55, P = 0.003). Moreover, in a joint analysis of SNPs rs9651118 and rs1801133, subjects with two unfavorable genotypes had a 1.76-fold increased risk of ARC compared with the reference group, and a statistically significant dose-response trend (P_trend_ = 0.001) was also observed. Further, in healthy controls and patients with cortical ARC, the allele T of SNP rs1801133 and the increasing number of unfavorable genotypes were significantly correlated with decreased MTHFR activity as well as increased tHcy levels. However, there was no significant association between FA, vitamins B12, B6 levels and *MTHFR* variants. Our data indicated that variants in *MTHFR* gene might individually and jointly influence susceptibility to ARC by affecting MTHFR enzyme activity and tHcy levels.

## Introduction

Cataract is the leading cause of visual impairment and blindness [1], affecting about 16 million people worldwide [2]. The most common type of cataract is age-related cataract (ARC), which is characterized by loss of transparency of the normal crystalline lens in people aged 50 years and over [3]. Although the etiology of ARC is not fully understood, it is widely accept that oxidative stress caused by excessive reactive oxygen species (ROS) generation plays a vital role in the development of ARC [4]. An increase in ROS can lead to DNA damage and formation of urea-insoluble proteins in the lens epithelium, and these processes are involved in cataract formation [4, 5].

Homocysteine (Hcy) is a crucial intermediate of methionine metabolism, and pathophysiological effects of Hcy involved the excessive production of ROS [6, 7]. Molecular studies have demonstrated that raised Hcy induced apoptosis by increasing ROS generation through activation of p38 MAPK [8], and down-regulating antioxidant enzymes such as heme oxygenase-1 and glutathione peroxidase [9, 10]. Additionally, elevated plasma total Hcy (tHcy) levels were found to be associated with increased risk of ARC in two clinical studies [11, 12]. Methylenetetrahydrofolate reductase (MTHFR) is the key enzyme for Hcy metabolism that catalyzes the conversion of 5, 10-methyltetrahydrofolate to 5-methyltetrahydrofolate, a methyl donor during the remethylation of Hcy [13]. More importantly, it has been reported that two variants (rs1801133 and rs1801131) in *MTHFR* gene might lead to a change of enzyme activity, which influenced the levels of tHcy [14, 15].

Taking all these considerations together, we speculated that functional SNPs in *MTHFR* could alter MTHFR enzyme activity, and thus might have an impact on Hcy metabolism, which further induced oxidative damage in lens and contributed to ARC risk. As Hcy metabolism also required substrates and cofactors such as folic acid (FA), vitamins B12 and B6, in our case-control study, we detected the plasma MTHFR activity, tHcy, FA, vitamins B12 and B6 levels, and performed a genotype-phenotype analysis of four potentially functional SNPs in *MTHFR* gene in Chinese population.

## Materials and Methods

### Study Populations

This case-control study involved 502 ARC patients and 890 healthy controls. All subjects were recruited from Zhongnan Hospital of Wuhan University and underwent a comprehensive ophthalmic examination, including visual acuity, slit-lamp biomicroscopy and ophthalmoscopic examination. ARC was defined as opacification of ocular lens resulting in best-corrected visual acuity (BCVA) less than 20/40 [16]. Lens opacities were classified into nuclear (LOCSIII score > 4), cortical (LOCSIII score > 2) and posterior subcapsular (PSC) (LOCSIII score > 2) types according to the Lens Opacities Classification System III (LOCSIII) [17]. The presence of more than one cataract type in at least one eye, or different pure types in both eyes was classified into the mixed type [18]. Patients with secondary cataract resulting from trauma, glaucoma, uveitis and other causes, or systemic disease such as diabetes, cancers, kidney diseases, cardiovascular and cerebrovascular diseases were excluded. The control group included subjects without cataract, other major eye diseases and major systemic diseases. Otherwise, we collected clinical information that have been associated with ARC risk, including smoking and drinking status, history of hypertension and fasting plasma glucose (FPG) levels (Table 1) [19–22]. Data about folate fortification (yes or no at the time of enrollment) and family history were also recorded. This study was approved by Ethnics Committee of Zhongnan Hospital of Wuhan University and met the declaration of Helsinki. All participants provided written informed consent accordingly and self-reported as ethnic Han Chinese.

**Table 1:** Clinical characteristics of study participants.

Group	N	Sex		Age		Smoking	Drinking	Hypertension	Blood Glucose	FA fortification	Family history
		Male (%)	Female (%)	Mean ± SD	Range	(%)	(%)	(%)	(mmol/L)	(%)	(%)
Control	890	424 (47.6)	466 (52.4)	67.1 ± 11.1	50–90	223 (25.1)	247 (27.8)	232 (26.1)	4.85 ± 0.50	20 (2.2)	-
ARC	502	231 (46.0)	271 (54.0)	70.2 ± 9.0 *	50–90	159 (31.7) *	154 (30.7)	173 (34.5) *	5.08 ± 0.53 *	11 (2.2)	12 (2.4)
Cortical	159	72 (45.3)	87 (54.7)	70.2 ± 9.4	50–90	52 (32.7)	51 (32.1)	54 (34.0)	5.07 ± 0.52	3 (1.9)	2 (1.3)
Nuclear	142	67 (47.2)	75 (52.8)	70.5 ± 8.4	50–86	38 (26.8)	39 (27.5)	42 (29.6)	5.08 ± 0.55	3 (2.1)	4 (2.8)
PSC	72	34 (47.2)	38 (52.8)	69.7 ± 8.2	53–89	27 (37.5)	23 (31.9)	31 (43.1)	5.07 ± 0.52	2 (2.8)	4 (4.2)
Mixed	129	58 (45.0)	71 (55.0)	70.2 ± 9.7	50–90	42 (32.6)	41 (31.8)	46 (35.7)	5.11 ± 0.52	3 (2.1)	3 (2.3)

Abbreviation: ARC, age-related cataract; PSC, posterior subcapsular; N, number; SD, standard deviation; FA, folic acid.

* P <0.05 in the comparisons between ARC patients and controls.

### Selection of SNPs and genotyping

Single nucleotide polymorphisms (SNPs) were chosen from the Hapmap database (http://hapmap.ncbi.nlm.nih.gov/, phase1, 2&3, Hapmap-CHB) and the NCBI dbSNP database (http://www.ncbi.nlm.nih.gov/snp/) based on the following criteria: (1) located in *MTHFR* gene region; (2) minor allele frequency (MAF) > 5%; (3) pairwise r^2^ ≤ 0.8; (4) predicted as a potentially functional SNP using a set of web-based SNP function prediction (FuncPred) program (http://snpinfo.niehs.nih.gov/snpinfo/snpfunc.htm) [23]. As a result, a total of 4 SNPs were selected (Table 2), which included one in potential miRNA-binding site (rs3737967), two non-synonymous SNPs (rs1801131, rs1801133) and one in potential transcription factor binding site (TFBS) (rs9651118).

**Table 2:** Characteristics of 4 SNPs in *MTHFR* gene.

SNP ID	Position ^a^	SNP type	Minor/Major allele	MAF (%) ^c^	HWE(P value) ^d^
rs3737967	Chr 1: 11787392	3’-UTR, miRNA-binding site ^b^	T/C	9.7	0.302
rs1801131	Chr 1: 11794419	Exon 7, Glu429Ala	C/A	18.3	0.630
rs1801133	Chr 1: 11796321	Exon 4, Ala222Val	T/C	41.6	0.259
rs9651118	Chr 1: 11802157	Intron 2, TFBS ^b^	C/T	33.5	0.216

Abbreviation: Chr, chromosome; 3’-UTR, 3’-untranslated region; Glu, glutamine; Ala, alanine; Val, valine; TFBS, transcription factor binding site; MAF, minor allele frequency; HWE, Hardy-Weinberg equilibrium.

^a^ Information for chromosome position is based on NCBI genome build 38.2.

^b^ MiRNA-binding site and TFBS were predicted by SNP function prediction (FuncPred) program.

^c^ MAF was calculated from the genotype data in our healthy controls.

^d^ The P value for HWE was calculaed from the genotype data in our healthy controls.

Genomic DNA was extracted from peripheral blood white cells using a phenol/chloroform method. SNPs were genotyped using a LightScanner 96 High Resolution Melt (HRM) system (Idaho Technology, Salt Lake City, UT, USA). PCR reaction for HRM was carried out in a volume of 10 μL containing 25 ng of genomic DNA, 5 pmol of each primer, 2 mmol dNTPs, 2 μL of 10×PCR buffer with 1.5 mmol/L MgCl_2_, 1 unit of Taq polymerase and 1 μL of LC green. After PCR reaction, HRM analysis was employed to thermally denature the small amplicons and measure the subtle differences in melting temperature (Tm) between different genotypes [24]. Heterozygous samples were identified by a change in melting curve shape, and different homozygotes were distinguished by Tm shifts (S1 Fig). For each SNP, the accuracy of genotyping was verified by DNA sequence analyses of 24 randomly selected samples (S2 Fig). Primer details for HRM and DNA sequence analyses, including primer sequences, product lengths and the annealing temperatures for each target, were shown in S1 Table.

### Determination of MTHFR activity, FA, vitamins B12, B6 and tHcy levels

Plasma samples were isolated by centrifugation (2000 *g* for 10 min at 4°C) and stored at -80°C until analyses. The tHcy levels were measured by an enzymatic assay [25] using the Hitachi 7020 automatic analyzer (Hitachi Medical Corp, Tokyo, Japan). This method has been confirmed over a linearity range of 1–65 μmol/L from plasma. The MTHFR activity, FA, vitamins B12 and B6 levels were detected by commercial enzyme-linked immunosorbent assay (ELISA) kits (Xinfan Biosystems, Shanghai, China) following the manufacturer's instructions. Briefly, 50 μL of the standard solutions or plasma samples were added into 96-well plates and incubated for 2 hours at 37°C. Then 50 μL of TMB substrate were pipetted into each well for 30 minutes at 37°C. After adding into the stop solutions (50 μL), the optical density (OD) values were determined at 450 nm wavelength using the Microplate Reader (Thermo Fisher Scientific, Waltham, MA, USA). The concentrations of MTHFR activity, FA, vitamins B12 and B6 were calculated based on the standard curves with detection limits of 20 U/L, 1 nmol/L, 30 pmol/L and 3 nmol/L, respectively. The intra-assay and inter-assay CV were 4.5% and 6.5% for tHcy, 5.2% and 7.3% for MTHFR activity, 7.5% and 8.9% for FA, 5.5% and 6.9% for vitamin B12 and 7.6% and 9.1% for vitamin B6, respectively.

### Statistical analyses

For clinical data, Pearson χ^2^ test and student t-test (or one-way ANOVA) were used to test for categorical variables and continuous variables, respectively. Hardy-Weinberg equilibrium (HWE) and allelic association analyses were assessed using Pearson χ^2^ test. Genotypic association analyses were assessed by logistic regression analyses under three models of inheritance (additive, dominant, recessive) after adjusting for age, sex, smoking and drinking status, history of hypertension, FPG levels, status of FA fortification and family history. When the samples were stratified, the χ^2^-based Q test was used to test the heterogeneity of effect sizes between different subgroups. The differences of MTHFR activity, tHcy, FA, vitamins B12 and B6 levels among ARC patients versus controls, and the effects of the *MTHFR* genotypes on MTHFR activity, tHcy, FA, vitamins B12 and B6 levels were analyzed by using analyses of covariance (ANCOVA) to adjust for covariates such as age, sex, smoking and drinking status, history of hypertension, FPG levels, status of FA fortification and family history. To control for multiple testing, the Bonferroni correction was applied. All these statistical analyses were performed in SPSS 17.0 software (SPSS Inc., Chicago, IL, USA)

The expectation-maximization (EM) algorithm was applied to derive the maximum-likelihood estimates of haplotype frequencies in the study population. The association between each common haplotype (>3%) and ARC risk was analyzed using the score statistics as implemented in Haplo Stats 1. 5. 0 program (Mayo Clinic, Rochester, MN, USA) [26]. This program could provide global score and haplotyple-specific score tests while allowing adjustment for covariates (age, sex, smoking and drinking status, history of hypertension, FPG levels, status of FA fortfication and family history). Power analysis was carried out using Power and Sample Size Program 3.0 (Vanderbilt University, Nashville, TN, USA).

## Results

### Population characteristics

Clinical characteristics of the study population were shown in Table 1. In the whole samples, there were significant differences in age, FPG levels, the rate of smoking and hypertension (P < 0.05) between the ARC and control groups. Within the ARC subtypes, age, sex, smoking and drinking status, the rate of hypertension, FPG levels and status of FA fortification showed no significant differences between different subtypes. The genotype distributions of all four SNPs were in accordance with HWE in the control group (P > 0.05, Table 2).

### Single-locus analyses

Among the 4 SNPs (Table 3), the minor alleles of SNPs rs1801133 (OR = 1.26, 95%CI = 1.08–1.48, P = 0.003) and rs9651118 (OR = 1.18, 95%CI = 1.00–1.39, P = 0.045) were significantly associated with increased odds of ARC. In genetic association analyses adjusted for covariates, significantly increased risks of ARC were found for SNP rs1801133 under both additive (OR = 1.25, 95%CI = 1.07–1.47, P = 0.006) and dominant models (OR = 1.44, 95%CI = 1.12–1.85, P = 0.004), and for SNP rs9651118 (OR = 1.32, 95%CI = 1.04–1.67, P = 0.020) assuming a dominant model. After the Bonferroni correction, the association between SNP rs1801133 and ARC risk remained significance (for allelic comparison, additive and dominant models, P_BON_ = 0.012, 0.024 and 0.016, respectively). Power analysis indicated that our population could provide 81.9% power (α = 0.05) to detect the association with an allelic OR of 1.26 for this polymorphism.

**Table 3:** Allelic and genotypic associations of 4 SNPs with ARC risk in our case-control study.

SNP	Genotype	Frequency (n)		Allelic comparison ^a^		Additive model ^a^		Dominant model ^a^		Recessive model ^a^	
		ARC (%)	Control (%)	OR (95%CI) ^b^	P ^b^/P_BON_ ^d^	OR (95%CI) ^c^	P ^c^/P_BON_ ^d^	OR (95%CI) ^c^	P ^c^/P_BON_ ^d^	OR (95%CI) ^c^	P ^c^
rs3737967	CC	410 (81.7)	729 (81.9)	1.08 (0.84–1.40)	0.555	1.10 (0.85–1.43)	0.460	1.09 (0.81–1.47)	0.575	1.46 (0.60–3.53)	0.401
C > T	CT	80 (15.9)	150 (16.9)								
	TT	12 (2.4)	11 (1.2)								
rs1801131	AA	334 (66.5)	596 (67.0)	1.06 (0.87–1.29)	0.554	1.06 (0.87–1.30)	0.563	1.03 (0.81–1.31)	0.814	1.37 (0.78–2.44)	0.277
A > C	AC	143 (28.5)	262 (29.4)								
	CC	25 (5.0)	32 (3.6)								
rs1801133	CC	139 (27.7)	312 (35.1)	**1.26 (1.08–1.48)**	**0.003/0.012**	**1.25 (1.07–1.47)**	**0.006/0.024**	**1.44 (1.12–1.85)**	**0.004/0.016**	1.25 (0.94–1.66)	0.121
C > T	CT	251 (50.0)	416 (46.7)								
	TT	112 (22.3)	162 (18.2)								
rs9651118	TT	195 (38.8)	402 (45.2)	1.18 (1.00–1.39)	0.045/0.180	1.18 (0.99–1.39)	0.054	1.32 (1.04–1.67)	0.020/0.080	1.09 (0.78–1.53)	0.621
T > C	TC	240 (47.8)	380 (42.7)								
	CC	67 (13.4)	108 (12.1)								

Abbreviation: OR (95%CI), odds ratio (95% confidence interval); ARC, age-related cataract.

^a^ In allelic and genotypic association analyses, the T, C, T and C alleles were regarded as risk alleles for SNPs rs3737967, rs1801131, rs1801133 and rs9651118, respectively.

^b^ P-value from Pearson ^2^ test of allele frequency.

^c^ P value from logistic regression after adjustment for age, sex, smoking and drinking status, history of hypertension, FPG levels, status of FA fortification and family history.

^d^ Multiple testing by the Bonferroni correction, P-value multiplied 4 (4 SNPs) to get a P_BON_ value.

Bold values were statistically significant after the Bonferroni correction.

When patients were stratified by the ARC subtypes (Table 4 and S2 Table), SNPs rs1801133 (OR = 1.44, 95%CI = 1.14–1.83, P = 0.003) and rs9651118 (OR = 1.29, 95%CI = 1.01–1.65, P = 0.044) showed significant allelic associations with the risk of cortical ARC. The genetic association analyses suggested that the dominant model provided the best fit for these two polymorphisms (the smallest P values) (for SNPs rs1801133 and rs9651118, OR = 2.01 and 1.50, 95%CI = 1.33–3.06 and 1.04–2.15, P = 0.001 and P = 0.028, respectively). After correction for multiple testing, only SNP rs1801133 was still significantly associated with the risk of cortical ARC (for allelic comparison, additive and dominant models, P_BON_ = 0.012, 0.040 and 0.004, respectively). Heterogeneity analysis indicated that there was a significant difference in effects of SNP rs1801133 on cortical ARC and other subtypes under a dominant model (P = 0.031). Otherwise, no significant differences were found for the allelic and genotypic association analyses of other two SNPs between the case and control groups (Table 3), even after stratifying by the ARC subtypes (S2 Table).

**Table 4:** Associations of SNPs rs1801133 and rs9651118 with the risk of ARC subtypes.

SNP	Control	Subtypes of ARC											
	(N = 890)	Cortical (N = 159)			Nuclear (N = 142)			PSC (N = 72)			Mixed (N = 129)		
	N (%)	N (%)	OR (95%CI) ^a^	P ^a^/P_BON_ ^b^	N (%)	OR (95%CI) ^a^	P ^a^	N (%)	OR (95%CI) ^a^	P ^a^	N (%)	OR (95%CI) ^a^	P ^a^
rs1801133													
C	1040 (58.4)	157 (49.4)	1 (Ref)		157 (55.3)	1 (Ref)		78 (54.2)	1 (Ref)		137 (53.1)	1 (Ref)	
T	740 (41.6)	161 (50.6)	**1.44 (1.14–1.83)**	**0.003/0.012**	127 (44.7)	1.14 (0.88–1.46)	0.319	66 (45.8)	1.19 (0.85–1.67)	0.319	121 (46.9)	1.24 (0.96–1.61)	0.106
CC	312 (35.1)	33 (20.8)	1 (Ref)		42 (29.6)	1 (Ref)		23 (31.9)	1 (Ref)		41 (31.8)	1 (Ref)	
CT + TT	578 (64.9)	126 (79.2)	**2.01 (1.33–3.06)**	**0.001/0.004**	100 (70.4)	1.34 (0.90–2.00)	0.154	49 (68.1)	1.13 (0.67–1.92)	0.650	88 (68.2)	1.19 (0.79–1.80)	0.401
Additive			**1.38 (1.08–1.76)**	**0.010/0.040**		1.14 (0.88–1.47)	0.320		1.11 (0.79–1.56)	0.564		1.22 (0.94–1.59)	0.136
rs9651118													
T	1184 (66.5)	193 (60.7)	1 (Ref)		180 (63.4)	1 (Ref)		85 (59.0)	1 (Ref)		172 (66.7)	1 (Ref)	
C	596 (33.5)	125 (39.3)	1.29 (1.01–1.65)	0.044/0.176	104 (36.6)	1.15 (0.88–1.49)	0.300	59 (41.0)	1.38 (0.98–1.95)	0.068	86 (33.3)	0.99 (0.75–1.31)	0.962
TT	402 (45.2)	57 (35.8)	1 (Ref)		56 (39.4)	1 (Ref)		26 (36.1)	1 (Ref)		56 (43.4)	1 (Ref)	
TC + TT	488 (54.8)	102 (64.2)	1.50 (1.04–2.15)	0.028/0.112	86 (60.6)	1.24 (0.85–1.80)	0.261	46 (63.9)	1.51 (0.90–2.53)	0.122	73 (56.6)	1.17 (0.79–1.72)	0.441
Additive			1.28 (0.99–1.64)	0.051		1.12 (0.86–1.46)	0.411		1.35 (0.95–1.92)	0.093		1.05 (0.79–1.40)	0.722

Abbreviation: N, number; ARC, age-related cataract; OR (95%CI), odds ratio (95% confidence interval); PSC,.posterior subcapsular; Ref, reference.

^a^ Allelic association analyses were assessed using Pearson ^2^ test, and genotypic association analyses were assessed by logistic regression after adjusting for age, sex, smoking and drinking status, history of hypertension, FPG levels, status of FA fortification and family history.

^b^ Multiple testing by the Bonferroni correction, P-value multiplied 4 (4 SNPs) to get a P_BON_ value.

Bold values were statistically significant after the Bonferroni correction.

### Haplotype analyses

Table 5 summarized the haplotype frequencies (≥3%) of four examined SNPs in cases and controls with the cumulative frequencies reaching 87.61% and 91.48%, respectively. A global score test showed significant difference in haplotype distributions between the case and control groups (P = 0.026). Haplotype "C-A-T-C", which exactly corresponded to the minor alleles of SNPs rs1801133 and rs9651118, was associated with an increased risk of ARC compared with the most common haplotye "C-A-C-T" (OR = 1.55, 95%CI = 1.16–2.07, P = 0.003). When patients were stratified by the ARC subtypes, similar associations were observed between haplotype "C-A-T-C" and the risks of cortical (OR = 1.97, 95%CI = 1.25–3.10, P = 0.003) and PSC (OR = 1.76, 95%CI = 1.08–3.16, P = 0.032) ARC. The significant associations for this haplotype with overall (P_BON_ = 0.021) and cortical ARC (P_BON_ = 0.021) risks remained after the Bonferroni correction.

**Table 5:** Associations of haplotypes in *MTHFR* gene with ARC risk.

Haplotype ^a^	Overall population						Cortical	Nuclear	PSC	Mixed
	ARC (%)	Control (%)	P_sim_ ^b^	OR (95%CI) ^c^	P ^c^/P_BON_ ^d^	Global test	OR (95%CI) ^c^	OR (95%CI) ^c^	OR (95%CI) ^c^	OR (95%CI) ^c^
C-A-C-T	23.06	23.85	0.180	1 (Ref)	0.171	^2^ = 15.88	1 (Ref)	1 (Ref)	1 (Ref)	1 (Ref)
C-A-T-T	21.57	23.31	0.821	1.02 (0.77–1.34)	0.812	df = 7	1.32 (0.81–2.15)	0.77 (0.47–1.26)	0.87 (0.44–1.73)	1.04 (0.68–1.58)
C-A-C-C	17.12	18.71	0.715	0.91 (0.75–1.35)	0.688	P = 0.026	1.29 (0.79–2.12)	0.91 (0.56–1.48)	1.16 (0.62–2.19)	0.82 (0.52–1.31)
C-A-T-C	15.10	10.70	0.005	**1.55 (1.16–2.07)**	**0.003/0.021**	P_sim_ ^b^ = 0.036	**1.97 (1.25–3.10)**	1.26 (0.78–2.05)	1.76 (1.08–3.16)	1.23 (0.78–1.95)
C-C-C-T	6.57	8.62	0.117	0.74 (0.56–1.15)	0.109		0.84 (0.40–1.81)	0.67 (0.33–1.36)	0.90 (0.38–1.91)	0.84 (0.46–1.56)
C-C-T-T	3.59	3.27	0.599	1.14 (0.73–1.78)	0.572		1.64 (0.91–3.05)	1.70 (0.74–3.79)	1.86 (0.66–4.15)	0.40 (0.15–1.87)
T-C-C-T	3.60	3.02	0.446	1.22 (0.78–1.92)	0.385		1.68 (0.75–2.75)	1.30 (0.62–2.71)	1.49 (0.54–4.13)	1.18 (0.50–2.67)

Abbreviation: ARC, age-related cataract; OR (95%CI), odds ratio (95% confidence interval); PSC,.posterior subcapsular; Ref,reference.

^a^ Alleles in haplotypes were presented in order of SNPs rs3737967, rs1801131, rs1801133 and rs9651118.

^b^ P_sim_ was calculated based on randomly permuting the trait and covariates and then computing the haplotype score statistics.

^c^ OR, 95%CI and P values were calculated after adjusting for age, sex, smoking and drinking status, history of hypertension, FPG levels, status of FA fortification and family history.

^d^ Multiple testing by the Bonferroni correction, P-value multiplied 7 (7 haplotypes) to get a P_BON_ value.

Bold values were statistically significant after the Bonferroni correction.

### Joint effect of unfavorable genotypes on ARC risk

To further assess the cumulative effect of the significant SNPs on ARC risk, a joint analysis of SNPs rs9651118 and rs1801133 was performed (Table 6). Assuming a dominant model, the unfavorable genotypes were defined as TC + CC for rs9651118 and CT + TT for rs1801133, respectively. Compared with the reference group, subjects with two unfavorable genotypes were at 1.76-fold increased risk of ARC (95%CI = 1.23–2.53, P = 0.002, P_BON_ = 0.006). A significant dose-response trend was also observed (P_trend_ = 0.001). After stratification by the ARC subtypes, subjects with one and two unfavorable genotypes had 2.73-fold (95%CI = 1.32–5.64, P = 0.007, P_BON_ = 0.021) increased risk and 3.89-fold (95%CI = 1.88–8.06, P < 0.001, P_BON_ = 0.001) increased risk of cortical ARC, respectively, in a dose-response manner (P_trend_ = 0.001).

**Table 6:** Joint effect of SNP rs1801133 and rs9651118 on ARC risk.

No. of unfavorable genotypes	Overall population				Cortical	Nuclear	PSC	Mixed
	ARC, N (%)	Control, N (%)	OR (95%CI) ^a^	P ^a^/P_BON_ ^b^	OR (95%CI) ^a^	OR (95%CI) ^a^	OR (95%CI) ^a^	OR (95%CI) ^a^
0	61 (29.5)	146 (70.5)	1 (Ref)		1 (Ref)	1 (Ref)	1 (Ref)	1 (Ref)
1	212 (33.4)	422 (66.6)	1.22 (0.85–1.74)	0.284	**2.73 (1.32–5.64)**	0.74 (0.43–1.27)	0.99 (0.46–2.13)	1.19 (0.66–2.14)
2	229 (41.6)	322 (58.4)	**1.76 (1.23–2.53)**	**0.002/0.006**	**3.89 (1.88–8.06)**	1.31 (0.78–2.22)	1.52 (0.71–3.23)	1.39 (0.76–2.54)
P _trend_			0.001		0.001	0.062	0.262	0.526

Abbreviation: ARC, age-related cataract; N, number; OR (95%CI), odds ratio (95% confidence interval); PSC,.posterior subcapsular; Ref, reference.

^a^ OR, 95%CI and P values were calculated after adjusting for age, sex, smoking and drinking status, history of hypertension, FPG levels, status of FA fortification and family history.

^b^ Multiple testing by the Bonferroni correction, P-value multiplied 3 (3 types of unfavorable genotypes) to get a P_BON_ value.

Bold values were statistically significant after the Bonferroni correction.

### MTHFR activity, FA, vitamins B12, B6 and tHcy levels

We calculated the required sample size for plasma tHcy and MTHFR measurement based on results from the Tan et al.'s [11] study which reported a mean difference of tHcy levels of 1.9μmol/L, a standard deviation of 4.8μmol/L in their population. Under the Type I error rate of 0.05 and a statistical power of 90%, 141 ARC patients and 141 healthy controls were randomly selected from the whole samples. This sample size could also provide > 90% power assuming a mean difference of MTHFR activity of ≥ 30 U/L and a standard deviation of ≤ 70 U/L in our population. The randomly selected subjects had similar clinical and genetic characteristics as compared to the whole samples (S3 Table).

In ANCOVA models adjusted for age, sex, smoking and drinking status, history of hypertension, FPG levels, status of FA fortification and family history, patients with ARC had lower activity of MTHFR (338.9 ± 35.8 U/L vs 369.6 ± 52.1 U/L, P < 0.001, Table 7) and higher levels of tHcy (12.3 ± 2.8 μmol/L vs 10.8 ± 1.9 μmol/L, P < 0.001, Table 7) than our control group. When patients were stratified by the ARC subtypes, MTHFR activity was significantly decreased, while tHcy levels were significantly increased in patients with cortical (for MTHFR, 316.3 ± 30.0 U/L vs 369.6 ± 52.1 U/L, P < 0.001; for tHcy, 13.4 ± 2.6 μmol/L vs 10.8 ± 1.9 μmol/L, P <0.001) and PSC (for MTHFR, 334.5 ± 32.5 U/L vs 369.6 ± 52.1 U/L, P = 0.003; for tHcy, 12.4 ± 1.8 μmol/L vs 10.8 ± 1.9 μmol/L, P = 0.002) ARC compared with those in the control group. The P values for heterogeneity of mean differences of MTHFR activity (P < 0.001) and tHcy levels (P = 0.001) were statistically significant between the associations observed for cortical ARC and other subtypes.

**Table 7:** MTHFR activity and tHcy levels with respect to different genotypes of SNP rs1801133 and No. of unfavorable genotypes in our population.

	Control		Overall ARC		Cortical		Nuclear		PSC		Mixed	
	N	Mean ± SD	N	Mean ± SD	N	Mean ± SD	N	Mean ± SD	N	Mean ± SD	N	Mean ± SD
MTHFR (U/L)												
Total	141	369.6 ± 52.1	141	338.9 ± 35.8 *	47	316.3 ± 30.0 *	38	356.9 ± 27.9	23	334.5 ± 32.5 *	33	353.4 ± 36.2
SNP rs1801133												
CC	53	391.0 ± 46.4	41	345.8 ± 34.3	12	338.7 ± 31.9	14	361.2 ± 33.2	8	333.4 ± 31.1	7	341.1 ± 39.6
CT	63	357.6 ± 49.3 **	72	333.7 ± 34.8	27	309.7 ± 27.2 **	18	348.6 ± 24.5	11	335.8 ± 35.1	16	356.0 ± 33.6
TT	25	354.5 ± 56.9 **	28	342.1 ± 39.2	8	304.8 ± 20.1 **	6	371.8 ± 16.4	4	333.1 ± 36.8	10	357.8 ± 39.6
CC + TT	88	356.7 ± 51.3 **	100	336.1 ± 36.1	35	308.6 ± 25.5 **	24	354.4 ± 24.7	15	335.1 ± 34.2	26	356.7 ± 35.3
Unfavorable genotypes (N)												
1	18	410.9 ± 43.5	16	337.7 ± 31.5	3	367.6 ± 10.8	4	345.7 ± 15.6	6	329.9 ± 33.6	3	325.9 ± 42.7
2	70	366.9 ± 53.3 **	53	341.1 ± 36.7	18	317.9 ± 32.3 **	15	360.8 ± 33.2	8	330.7 ± 26.3	12	356.8 ± 34.3
3	53	359.2 ± 46.9 **	72	337.6 ± 36.4	26	309.2 ± 23.9 **	19	356.2 ± 25.6	9	340.9 ± 38.9	18	355.7 ± 36.6
tHcy (μmol/L)												
Total	141	10.8 ± 1.9	141	12.3 ± 2.8 *	47	13.4 ± 2.6 *	38	11.5 ± 2.5	23	12.4 ± 1.8 *	33	11.6 ± 2.4
SNP rs1801133												
CC	53	10.1 ± 1.4	41	11.8 ± 2.6	12	11.3 ± 2.7	14	11.8 ± 2.8	8	12.6 ± 2.5	7	11.8 ± 2.7
CT	63	11.1 ± 2.1 **	72	12.6 ± 2.3	27	13.8 ± 2.2 **	18	11.2 ± 2.5	11	12.4 ± 1.0	16	12.3 ± 2.0
TT	25	11.5 ± 1.8 **	28	12.2 ± 2.8	8	15.0 ± 2.1 **	6	11.3 ± 1.9	4	12.3 ± 2.2	10	10.4 ± 2.4
CT + TT	88	11.2 ± 2.0 **	100	12.5 ± 2.4	35	14.1 ± 2.2 **	24	11.3 ± 2.4	15	12.4 ± 1.3	26	11.6 ± 2.3
Unfavorable genotypes (N)												
0	18	9.6 ± 0.8	16	12.0 ± 2.5	3	10.3 ± 2.0	4	13.0 ± 2.2	6	12.0 ± 2.5	3	12.4 ± 3.8
1	70	10.8 ± 1.8 **	53	12.0 ± 2.5	18	12.8 ± 2.7 **	15	10.9 ± 2.5	8	12.8 ± 1.7	12	11.2 ± 2.1
2	53	11.2 ± 2.1 **	72	12.6 ± 2.5	26	14.1 ± 2.3 **	19	11.6 ± 2.6	9	12.4 ± 1.4	18	11.7 ± 2.4

Abbreviation: N, number; ARC, age-related cataract; PSC, posterior subcapsular; SD, standard deviation.

* P < 0.05, in the comparisons between ARC patients and healthy controls.

** P < 0.05, in the comparisons between different genotypes of SNP rs1801133 and in the comparisons between different numbers of unfavorable genotypes (SNPs rs1801133 + rs9651118)

Otherwise, FA levels were significantly decreased in patients with ARC (15.7 ± 3.0 nmol/L vs 16.3 ± 1.7 nmol/L, P = 0.008) and especially in patients with cortical (14.9 ± 2.8 nmol/L vs 16.3 ± 1.7 nmol/L, P = 0.001) and PSC subtypes (15.1 ± 3.8 nmol/L vs 16.3 ± 1.7 nmol/L, P = 0.010) as compared to those in the control group (S4 Table). Although there were no significant difference in vitamin B12 levels between the case and control groups, in the subgroup analyses stratified by the ARC subtypes, patients with cortical ARC had lower levels of vitamin B12 than our healthy controls (254.3 ± 36.2 pmol/L vs 271.9 ± 37.4 pmol/L, P = 0.011) (S5 Table). However, no significant difference was found for levels of vitamin B6 between the case and control groups, even after stratifying by the ARC subtypes (S6 Table).

### Associations of *MTHFR* gene variants with MTHFR activity, FA, vitamins B12, B6 and tHcy levels

**Fig 1:**
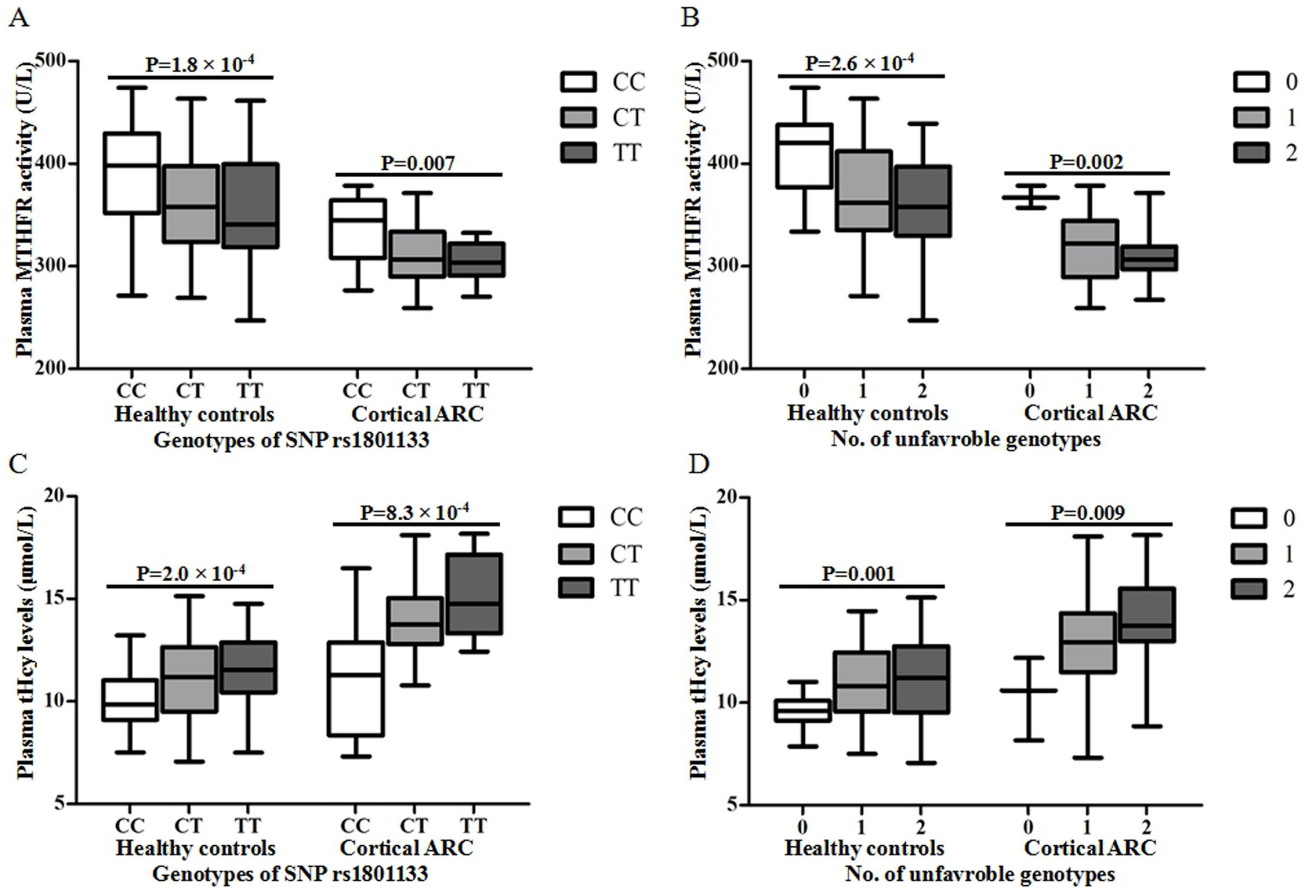
Associations of SNP rs1801133 and No. of unfavorable genotypes with the plasma MTHFR activity and tHcy levels in healthy controls and patients with cortical ARC. (A) Associations of SNP rs1801133 with MTHFR activity, (B) Associations of No. of unfavorable genotypes with MTHFR activity, (C) Associations of SNP rs1801133 with tHcy levels, (D) Associations of No. of unfavorable genotypes with tHcy levels. ANCOVA models were used to assess statistical significance. In Fig 1 (A) and (C), associations of SNP rs1801133 with the plasma MTHFR activity and tHcy levels were calculated based on an additive model.

Results from ANCOVA (Fig 1 and Table 7) models indicated that the minor allele T of SNP rs1801133 was significantly associated with decreased MTHFR activity and increased tHcy levels under both additive and dominant models in healthy controls (for MTHFR activity and tHcy levels under both additive and dominant models, P < 0.001) and patients with cortical ARC (for MTHFR activity under an additive model and a dominant model, P = 0.007 and P < 0.001, respectively; for tHcy levels under both additive and dominant models, P < 0.001). Although SNP rs9651118 was not associated with MTHFR activity and tHcy levels (S7 Table), a joint analysis of SNPs rs9651118 and rs1801133 indicated that there were significant trends of decreasing MTHFR activity and increasing tHcy levels with increasing number of unfavorable genotypes in healthy controls (for MTHFR activity and tHcy levels, P <0.001 and P = 0.001, respectively) and patients with cortical ARC (for MTHFR activity and tHcy levels, P = 0.002 and 0.009, respectively). However, there were no significant associations of *MTHFR* variants with the plasma levels of folic acid, vitamins B12 and B6, even in the subgroup analyses stratified by the ARC subtypes (S4–S6 Tables).

## Discussion

Plasma tHcy levels and variants in MTHFR gene, which have been implicated in the pathogenesis of cancers [27], vitiligo [28], cardiovascular [29, 30] and cerebrovascular [31, 32] diseases, was also reported to be correlated with several age-related eye diseases such as age-related macular degeneration (AMD) [33], retinal vein occlusion (RVO) [34] and primary open-angle glaucoma (POAG) [35]. In the present study, we examined associations of four potentially functional SNPs in *MTHFR* gene with ARC risk, and detected the plasma MTHFR activity, tHcy, FA, vitamins B12 and B6 levels in Chinese population. Accumulated evidence has suggested that Hcy might be a cataractogenic stressor through a multistep process. First, higher levels of Hcy altered the cellular redox state [36], and further caused the accumulation of unfolded and misfolded proteins [37], which could trigger the sustained unfolded protein response (UPR) [38]. Then, uncontrolled URP could induce the excessive production of ROS through the URP-regulated oxidative protein folding machinery in the endoplasmic reticulum and the mitochondria [39], which eventually resulted in cell death and apoptosis in lens epithelial cells. MTHFR is the main regulatory enzyme for Hcy metabolism, and its activity is crucial for Hcy homeostasis. SNP rs1801133 is a C > T missense mutation in *MTHFR* gene that lead to an amino acid change from alanine to valine at codon position 222 of MTHFR protein. This amino acid change is at the bottom of (βα)_8_ barrel in the N-terminal catalytic domain of the protein, which plays a vital role in MTHFR structure and enzyme activity [40, 41]. Our results showed that the minor allele T of SNP rs1801133 had an adverse effect on ARC risk, especially on cortical ARC risk. Patients with ARC had lower activity of MTHFR and higher levels of tHcy. Further genotype-phenotype analysis indicated that the T allele was also associated with decreased MTHFR activity and increased tHcy levels in healthy controls and patients with cortical ARC. These findings supported our hypothesis that potentially functional SNP in *MTHFR* gene might involve in Hcy metabolism and ARC development.

The stronger associations with cortical ARC than with other subtypes might come from several reasons. Although oxidative damage contributed to the formation of cortical, PSC and nuclear ARC, the manner in which it did was different [42]. The lens cortex, being located in the outer zone of the lens, was probably more susceptible to extraneous oxidative insults transmitted through the aqueous humor than the deeply situated nucleus [42]. PSC cataract formation from epithelial cells of the posterior lens capsule could occur in a similar way [11]. However, genetic contributions among cortical and PSC cataract might be distinct. Heritability for cortical cataract in several studies was estimated to be 24–58% [43–45], while that for PSC cataract was not statistically significant [45]. In our study, we found significantly decreased MTHFR activity and increased tHcy levels in PSC ARC patients compared with those in healthy controls. However, only haplotype "C-A-T-C" showed a nominally significant association with PSC ARC risk. These findings were consistent with previous report [11] and partially supported the view mentioned above.

Another SNP, rs9651118, was nominally associated with overall and cortical ARC risks in our study. Further in haplotype analyses, only one haplotype (C-A-T-C) harboring both minor alleles of SNPs rs9651118 and rs1801133 was related to an increased risk of ARC, suggesting the potential existence of locus-to-locus interaction. To further assess the joint effect of SNPs rs9651118 and rs1801133 on ARC risk, a combination analysis of these two variants was carried out, and the results indicated that there was a significant trend of increasing ARC risk with increasing number of unfavorable genotypes, reinforcing the results of haplotype analyses. In addition, from a biological perspective, besides the potential impact of exonic SNP rs1801133 on MTHFR enzyme activity, it should also be noted that the intronic SNP rs9651118 was predicted to be at a TFBS of the gene that might involve in the stability of DNA molecule [46] and gene regulatory networks [47]. By searching the Blood eQTL Browser database (http://genenetwork.nl/bloodeqtlbrowser/) [48], we even found that the minor allele C of SNP rs9651118, which increased ARC risk in our study, was also significantly associated with lower *MTHFR* mRNA expression levels. This evidence further raised the possibility that this SNP is involved in gene regulation. In current study, SNP rs9651118 was not correlated with the plasma MTHFR activity and tHcy levels. However, we found dose-response effects of the numbers of the unfavorable genotypes on the plasma MTHFR activity and tHcy levels in healthy controls and patients with cortical ARC. Combining all these factors together, it is reasonable to speculate that the interaction of multiple SNPs in *MTHFR* gene might contribute to the pathogenesis of ARC in Chinese Han population.

SNP rs1801131 is at codon 429 resulting in a glutamine (Glu) to alanine (Ala) substitution which occurs in the C-terminal regulatory domain of MTHFR protein. This domain mainly stabilizes the catalytic domain [49] and partially mediates NADPH binding [50], but does not greatly regulate the enzyme activity [50]. Both *in vivo* [15] and *in vitro* [51] studies indicated that the activity of the Glu429Ala protein was clearly higher than that observed with SNP rs1801133 C > T mutation, suggesting that Hcy metabolism might not be significantly disrupted by SNP rs1801131 alone [51]. Our results also demonstrated that SNP rs1801131 was not correlated with the plasma MTHFR activity, tHcy levels, and ARC risk. However, we noticed that an Estonian study reported a significant association of SNP rs1801131 with the risk of mixed cataract [52]. To our knowledge, the MAF of SNP rs1801133 in our control group was 18.3%, which was similar to that in HapMap CHB database (19.0%), but significantly different from those in Estonian Caucasians (29.7%) and HapMap CEU database (34.1%), suggesting that the ethnic difference of this variant did exist. Apart from genetic background, the exclusion criteria for ARC patients were also different. To avoid the selection bias, individuals with secondary cataract and major systematic diseases were all excluded in our study, while Zetterberg *et al* [52] did not consider the influence of systematic diseases. This might also contribute to the discrepancies between the two studies.

Interpretation of our present study should also consider the following limitations. First, although we adjusted for several risk factors of ARC, other confounding factors that were not collected in our study might also cause the bias. Second, in our study, the relatively small number of patients with PSC ARC resulted in low power to detect an association. Finally, our healthy controls were definitely younger than the ARC group, which meant that some of these young controls may develop ARC in the future. Although we replicated the current results when we performed a stratification analysis between our old controls (> an average age of 67.1) and ARC patients (data not shown).

In conclusion, our data suggested that variants in *MTHFR* gene might individually and jointly influence susceptibility to ARC by affecting MTHFR enzyme activity and tHcy levels. These findings need to be replicated by larger studies, and functional studies are warranted to elucidate these effects.

## Supporting Information

S1 Dataset Clinical and genetic data in our population. (XLS)

S2 Dataset Clinical and genetic data of participants that were randomly selected to measure MTHFR activity and tHcy levels. (XLS)

S1 Fig HRM plots for different genotypes of four SNPs. The normalized melting peaks are given in the left column, and the normalized melting curves are given in the right column. Arrows indicate the genotypes. The representative HRM plots of SNP rs3737967, rs1801131, rs1801133 and rs9651118 are shown in A, B, C, and D, respectively.
(TIF)

S2 Fig DNA sequence analysis for different genotypes of four SNPs. The three genotypes of SNPs rs3737967 (C > T), rs1801131 (A > C), rs1801133 (C > T) and rs9651118 (T > C) are shown in A, B, C, and D, respectively.
(TIF)

S1 Table Primer details for HRM and DNA sequence analyses in our study. (DOC)

S2 Table Associations of SNPs rs3737967 and rs1801131 with the risk of ARC subtypes. (DOC)

S3 Table Comparative analyses of clinical and genetic characteristics between the randomly selected subjects and the whole samples. (DOC)

S4 Table Associations of *MTHFR* variants with plasma FA levels in our population. (DOC)

S5 Table Associations of *MTHFR* variants with plasma vitamin B12 levels in our population. (DOC)

S6 Table Associations of *MTHFR* variants with plasma vitamin B6 levels in our population. (DOC)

S7 Table MTHFR activity and tHcy levels with respect to different genotypes of SNPs rs3737967, rs1801131 and rs9651118 in healthy controls and different types of ARC. (DOC)
