# Supplementary figures and images for: Associations of Polymorphisms in MTHFR Gene with the Risk of Age-Related Cataract in Chinese Han Population: A Genotype-Phenotype Analysis
Source: PLoS One. 2015 Dec 21;10(12):e0145581. doi: 10.1371/journal.pone.0145581 (PMC4686960; doi:10.1371/journal.pone.0145581)

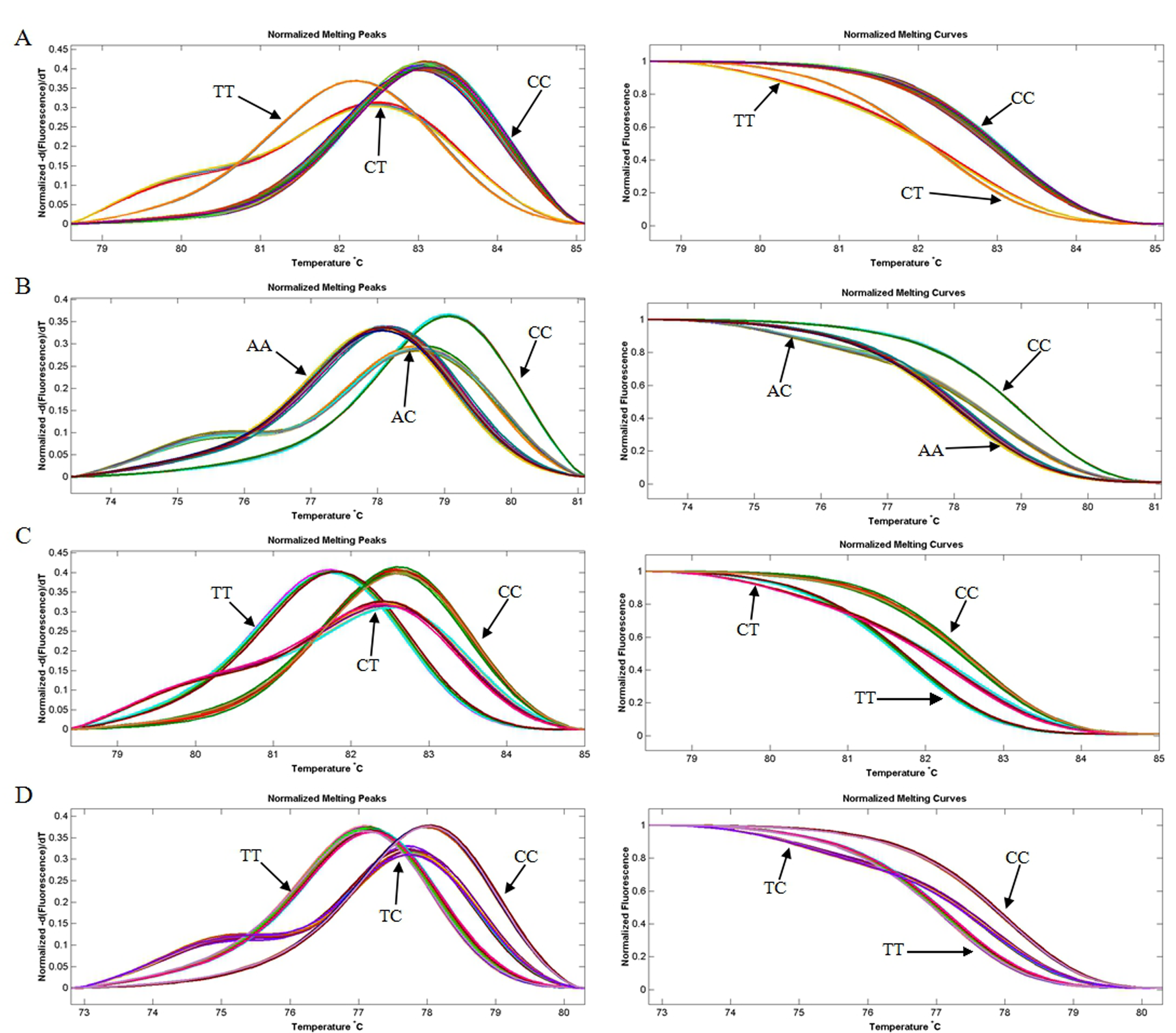

Supplement: S1 Fig — The normalized melting peaks are given in the left column, and the normalized melting curves are given in the right column. Arrows indicate the genotypes. The representative HRM plots of SNP rs3737967, rs1801131, rs1801133 and rs9651118 are shown in A, B, C, and D, respectively. (TIF) [file pone.0145581.s003.tif]

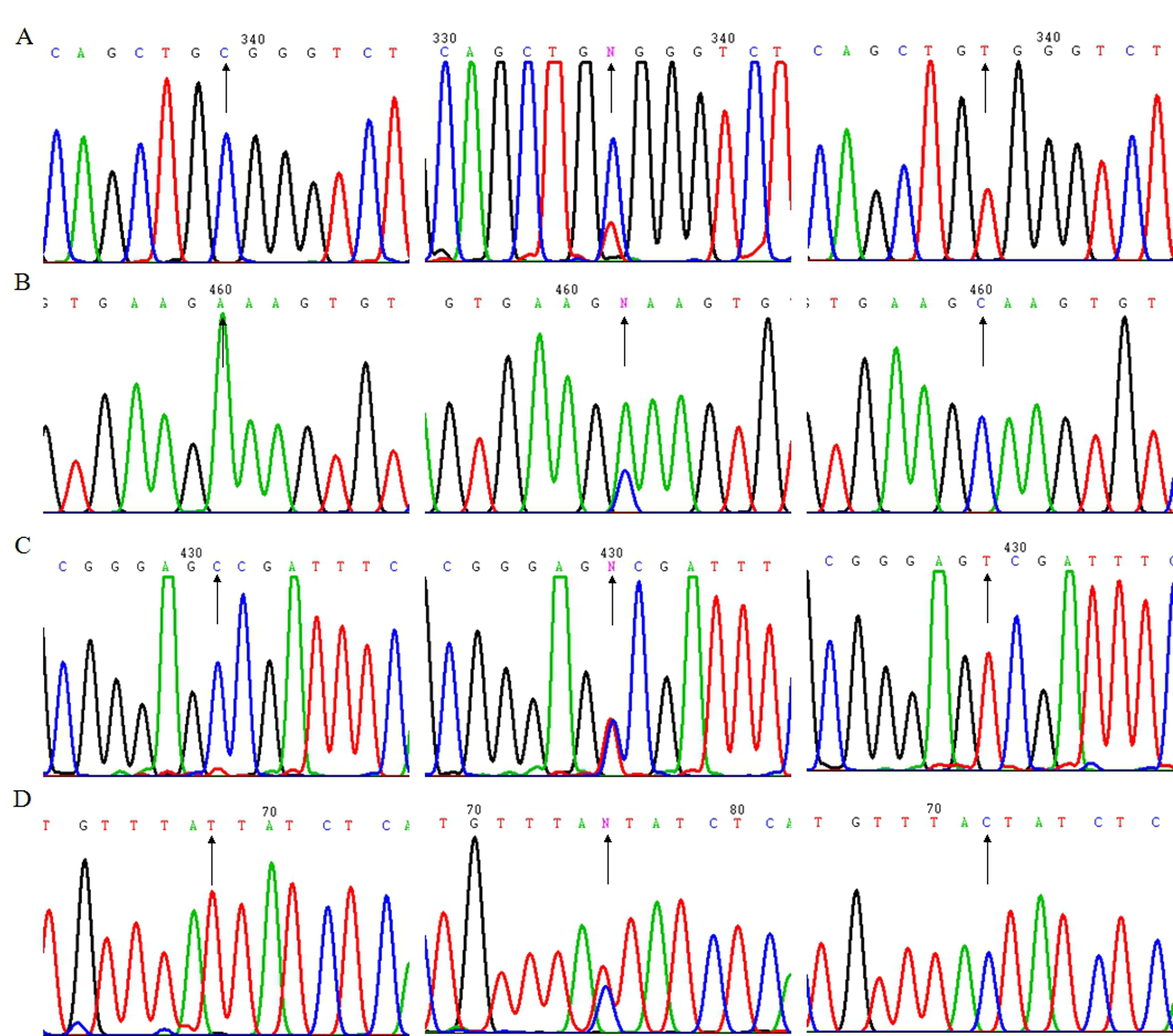

Supplement: S2 Fig — The three genotypes of SNPs rs3737967 (C > T), rs1801131 (A > C), rs1801133 (C > T) and rs9651118 (T > C) are shown in A, B, C, and D, respectively. (TIF) [file pone.0145581.s004.tif]
