# Supplementary material for: Associations of Polymorphisms in MTHFR Gene with the Risk of Age-Related Cataract in Chinese Han Population: A Genotype-Phenotype Analysis
Source: PLoS One. 2015 Dec 21;10(12):e0145581. doi: 10.1371/journal.pone.0145581 (PMC4686960; doi:10.1371/journal.pone.0145581)
Supplement: S1 Table — (DOC) [file pone.0145581.s005.doc]

| **S1 Table. Primer details for HRM and DNA sequence analyses in our study..** | | | | |
| --- | --- | --- | --- | --- |
| Method | SNP | Primer | Length (bp) | Ann. Temp (°C) |
| HRM | rs3737967 | Forward: GCTCTGTGGCCTGTCTC | 48 | 57 |
|  |  | Reverse: TCTGGCTATTCCTTCAGACCC |  |  |
|  | rs1801131 | Forward: GGAGGAGCTGACCAGTGA | 50 | 57 |
|  |  | Reverse: GGTAAAGAACGAAGACTTCAAAGACA |  |  |
|  | rs1801133 | Forward: GACCTGAAGCACTTGAAGGA | 58 | 59 |
|  |  | Reverse: GCTGCGTGATGATGAAATCG |  |  |
|  | rs9651118 | Forward: AGACTTTTCACAGCGCTT | 53 | 54 |
|  |  | Reverse: ATGATGTCTTAACTCACCTGAGAT |  |  |
| Sequencing | rs3737967 | Forward: TGCTGAGCTTAGCCTTGTGC | 574 | 57 |
|  |  | Reverse: GTGCTCCGGGAACTGACTG |  |  |
|  | rs1801131 | Forward: GCTCCCTCTAGCCAATCCCT | 577 | 61 |
|  |  | Reverse: CCACTCCAGCATCACTCACTTTG |  |  |
|  | rs1801133 | Forward: ATGTTTAATCCGGTGCCTAG | 564 | 59 |
|  |  | Reverse: CAAGTGATGCCCATGTCG |  |  |
|  | rs9651118 | Forward: ATATGTGAGGTGCCGTTAT | 596 | 56 |
|  |  | Reverse: CACTGTATTCTTTGAGGGA |  |  |
| Abbreviation: HRM, High Resolution Melt; Ann. Temp., annealing temperature. | | | | |
