# Supplementary material for: Associations of Polymorphisms in MTHFR Gene with the Risk of Age-Related Cataract in Chinese Han Population: A Genotype-Phenotype Analysis
Source: PLoS One. 2015 Dec 21;10(12):e0145581. doi: 10.1371/journal.pone.0145581 (PMC4686960; doi:10.1371/journal.pone.0145581)
Supplement: S2 Table — (DOC) [file pone.0145581.s006.doc]

| **S2 Table. Associations of SNPs rs3737967 and rs1801131 with the risk of ARC subtypes.** | | | | | | | | | | | | | | | | |
| --- | --- | --- | --- | --- | --- | --- | --- | --- | --- | --- | --- | --- | --- | --- | --- | --- |
| Model | Control | Subtypes of ARC | | | | | | | | | | | | | | |
|  | (N=890) | Cortical (N=159) | | |  | Nuclear (N=142) | | |  | PSC (N=72) | | |  | Mixed (N=129) | | |
|  | N (%) | N (%) | OR (95%CI) ^a^ | P ^a^ |  | N (%) | OR (95%CI) ^a^ | P ^a^ |  | N (%) | OR (95%CI) ^a^ | P ^a^ |  | N (%) | OR (95%CI) ^a^ | P ^a^ |
| rs3737967 |  |  |  |  |  |  |  |  |  |  |  |  |  |  |  |  |
| C | 1608 (90.3) | 281 (88.4) | 1 (Ref) |  |  | 256 (90.1) | 1 (Ref) |  |  | 129 (89.6) | 1 (Ref) |  |  | 234 (90.7) | 1 (Ref) |  |
| T | 172 (9.7) | 37 (11.6) | 1.23 (0.84-1.80) | 0.279 |  | 28 (9.9) | 1.02 (0.67-1.56) | 0.917 |  | 15 (10.4) | 1.09 (0.62-1.90) | 0.769 |  | 24 (9.3) | 0.96 (0.61-1.50) | 0.854 |
| CC | 729 (81.9) | 128 (80.5) | 1 (Ref) |  |  | 116 (81.7) | 1 (Ref) |  |  | 59 (81.9) | 1 (Ref) |  |  | 107 (82.9) | 1 (Ref) |  |
| CT + TT | 161 (18.1) | 31 (19.5) | 1.19 (0.80-1.86) | 0.430 |  | 26 (18.3) | 1.15 (0.72-1.83) | 0.571 |  | 13 (18.1) | 1.03 (0.53-2.00) | 0.926 |  | 22 (17.1) | 0.97 (0.57-1.63) | 0.896 |
| Additive |  |  | 1.26 (0.87-1.83) | 0.215 |  |  | 1.11 (0.73-1.69) | 0.617 |  |  | 1.00 (0.56-1.78) | 0.997 |  |  | 0.98 (0.62-1.55) | 0.919 |
| rs1801131 |  |  |  |  |  |  |  |  |  |  |  |  |  |  |  |  |
| A | 1454 (81.7) | 254 (79.9) | 1 (Ref) |  |  | 227 (79.9) | 1 (Ref) |  |  | 114 (79.2) | 1 (Ref) |  |  | 216 (83.7) | 1 (Ref) |  |
| C | 326 (18.3) | 64 (20.1) | 1.12 (0.83-1.52) | 0.444 |  | 57 (20.1) | 1.12 (0.82-1.53) | 0.480 |  | 30 (20.8) | 1.17 (0.77-1.79) | 0.454 |  | 42 (16.3) | 0.87 (0.61-1.23) | 0.427 |
| AA | 596 (67.0) | 103 (64.8) | 1 (Ref) |  |  | 90 (63.4) | 1 (Ref) |  |  | 49 (68.1) | 1 (Ref) |  |  | 92 (71.3) | 1 (Ref) |  |
| AC + CC | 294 (33.0) | 56 (35.2) | 1.11 (0.77-1.60) | 0.574 |  | 52 (36.6) | 1.16 (0.79-1.69) | 0.458 |  | 23 (31.9) | 0.96 (0.56-1.64) | 0.879 |  | 37 (28.7) | 0.78 (0.51-1.20) | 0.245 |
| Additive |  |  | 1.13 (0.83-1.53) | 0.448 |  |  | 1.12 (0.81-1.55) | 0.495 |  |  | 1.13 (0.74-1.74) | 0.570 |  |  | 0.82 (0.57-1.18) | 0.288 |
| Abbreviation: OR (95%CI)-odds ratio (95% confidence interval); N, number; .ARC, age-related cataract; PSC, posterior subcapsular; Ref, reference.  ^a^ Allelic association analyses were assessed using Pearson χ^2^ test, and genotypic association analyses were assessed by logistic regression after adjusting for age, sex, smoking and drinking status, history of hypertension, FPG levels, status of FA fortification and family history. | | | | | | | | | | | | | | | | |
