# Supplementary material for: Associations of Polymorphisms in MTHFR Gene with the Risk of Age-Related Cataract in Chinese Han Population: A Genotype-Phenotype Analysis
Source: PLoS One. 2015 Dec 21;10(12):e0145581. doi: 10.1371/journal.pone.0145581 (PMC4686960; doi:10.1371/journal.pone.0145581)
Supplement: S3 Table — (DOC) [file pone.0145581.s007.doc]

| **S3 Table. Comparative analyses of clinical and genetic characteristics between the randomly selected subjects and the whole samples.** | | | | | | | |
| --- | --- | --- | --- | --- | --- | --- | --- |
| Variables | Control | | |  | ARC | | |
|  | Selected subjects (N=141) | Whole samples (N=650) | P ^a^ |  | Selected subjects (N=141) | Whole samples (N=502) | P ^a^ |
| Male, n (%) | 68 (48.2) | 424 (47.6) | 0.897 |  | 59 (41.8) | 231 (46.0) | 0.379 |
| Smoking, n (%) | 36 (25.5) | 223 (25.1) | 0.892 |  | 36 (25.5) | 159 (31.7) | 0.161 |
| Drinking, n (%) | 37 (26.2) | 247 (27.8) | 0.709 |  | 37 (26.2) | 154 (30.7) | 0.308 |
| Hypertension, n (%) | 33 (23.4) | 232 (26.1) | 0.501 |  | 50 (36.5) | 173 (34.5) | 0.826 |
| FPG, mmol/L | 4.79 ± 0.50 | 4.85 ± 0.50 | 0.158 |  | 5.14 ± 0.51 | 5.08 ± 0.53 | 0.272 |
| Rs3737967, n (%) ^b^ |  |  | 0.762 |  |  |  | 0.871 |
| CC | 27 (19.1) | 161 (18.1) |  |  | 25 (17.7) | 92 (18.3) |  |
| CT + TT | 114 (80.9) | 729 (81.9) |  |  | 116 (82.3) | 410 (81.7) |  |
| Rs1801131, n (%) ^b^ |  |  | 0.445 |  |  |  | 0.326 |
| AA | 42 (29.8) | 294 (33.0) |  |  | 41 (29.1) | 168 (33.5) |  |
| AC + CC | 99 (70.2) | 596 (67.0) |  |  | 100 (70.9) | 334 (66.5) |  |
| Rs1801133, n (%) |  |  | 0.841 |  |  |  | 0.817 |
| TT | 53 (37.6) | 312 (35.1) |  |  | 41 (29.1) | 139 (27.7) |  |
| CT | 63 (44.7) | 416 (46.7) |  |  | 72 (51.1) | 251 (50.0) |  |
| CC | 25 (17.7) | 162 (18.2) |  |  | 28 (19.8) | 112 (22.3) |  |
| Rs9651118, n (%) |  |  | 0.200 |  |  |  | 0.225 |
| TT | 53 (37.6) | 402 (45.2) |  |  | 44 (31.2) | 195 (38.8) |  |
| TC | 71 (50.3) | 380 (42.7) |  |  | 78 (55.3) | 240 (47.8) |  |
| CC | 17 (12.1) | 108 (12.1) |  |  | 19 (13.5) | 67 (13.4) |  |
| No. Of unfavorable genotypes, n (%) |  |  | 0.547 |  |  |  | 0.514 |
| 0 | 18 (12.8) | 146 (16.4) |  |  | 16 (11.3) | 61 (12.2) |  |
| 1 | 70 (49.6) | 422 (47.4) |  |  | 53 (37.6) | 212 (42.2) |  |
| 2 | 53 (37.6) | 322 (36.2) |  |  | 72 (51.1) | 229 (45.6) |  |
| Abbreviation: N, number; ARC, age-related cataract; FPG, fasting plasma glucose.  ^a^ Pearson χ^2^ test and student t-test were used to test for categorical variables and continuous variables, respectively. ^b^ For SNPs rs3737967 and rs1801131, the TT and CC carriers were relatively rare in our randomly selected subjects, so we provided the results of comparative analyses under a dominant model. | | | | | | | |
