# Supplementary material for: Associations of Polymorphisms in MTHFR Gene with the Risk of Age-Related Cataract in Chinese Han Population: A Genotype-Phenotype Analysis
Source: PLoS One. 2015 Dec 21;10(12):e0145581. doi: 10.1371/journal.pone.0145581 (PMC4686960; doi:10.1371/journal.pone.0145581)
Supplement: S7 Table — (DOC) [file pone.0145581.s011.doc]

| **S7 Table.** **Associations of SNPs rs3737967 and rs1801131 with MTHFR activity and tHcy levels in our population.** | | | | | | | | | | | | | | | | | |
| --- | --- | --- | --- | --- | --- | --- | --- | --- | --- | --- | --- | --- | --- | --- | --- | --- | --- |
|  | Control | |  | Overall ARC | |  | Cortical | |  | Nuclear | |  | PSC | |  | Mixed | |
|  | N | Mean ± SD |  | N | Mean ± SD |  | N | Mean ± SD |  | N | Mean ± SD |  | N | Mean ± SD |  | N | Mean ± SD |
| MTHFR (U/L) |  |  |  |  |  |  |  |  |  |  |  |  |  |  |  |  |  |
| SNP rs3737967 ^a^ |  |  |  |  |  |  |  |  |  |  |  |  |  |  |  |  |  |
| CC | 114 | 368.1 ± 51.2 |  | 116 | 337.5 ± 35.5 |  | 41 | 315.7 ± 30.9 |  | 30 | 353.5 ± 27.3 |  | 18 | 333.6 ± 31.5 |  | 27 | 355.5 ± 35.2 |
| CT + TT | 27 | 375.6 ± 56.1 |  | 25 | 345.4 ± 36.9 |  | 6 | 320.3 ± 24.9 |  | 8 | 369.9 ± 27.9 |  | 5 | 337.8 ± 39.3 |  | 6 | 344.0 ± 42.5 |
| SNP rs1801131 ^a^ |  |  |  |  |  |  |  |  |  |  |  |  |  |  |  |  |  |
| AA | 99 | 364.8 ± 53.1 |  | 100 | 339.7 ± 35.5 |  | 36 | 320.1 ± 29.9 |  | 25 | 358.8 ± 28.8 |  | 13 | 332.5 ± 28.7 |  | 26 | 353.4 ± 36.9 |
| AC + CC | 42 | 380.6 ± 48.5 |  | 41 | 337.0 ± 36.8 |  | 11 | 303.8 ± 28.2 |  | 13 | 353.3 ± 26.7 |  | 10 | 337.1 ± 38.3 |  | 7 | 353.3 ± 36.1 |
| SNP rs9651118 |  |  |  |  |  |  |  |  |  |  |  |  |  |  |  |  |  |
| TT | 53 | 372.6 ± 59.8 |  | 44 | 335.7 ± 33.7 |  | 12 | 322.0 ± 38.6 |  | 9 | 346.8 ± 18.2 |  | 12 | 328.1 ± 28.9 |  | 11 | 350.0 ± 37.8 |
| TC | 71 | 368.3 ± 46.7 |  | 78 | 341.0 ± 37.1 |  | 27 | 313.0 ± 28.6 |  | 24 | 361.6 ± 28.3 |  | 8 | 340.6 ± 37.7 |  | 19 | 354.7 ± 34.4 |
| CC | 17 | 365.9 ± 50.1 |  | 19 | 337.6 ± 36.0 |  | 8 | 318.6 ± 20.6 |  | 5 | 352.5 ± 39.0 |  | 3 | 343.8 ± 38.6 |  | 3 | 357.3 ± 54.9 |
| TC + CC | 88 | 367.8 ± 47.1 |  | 97 | 340.3 ± 36.7 |  | 35 | 314.3 ± 26.8 |  | 29 | 360.1 ± 29.8 |  | 11 | 341.5 ± 36.0 |  | 22 | 353.4 ± 36.2 |
| tHcy (μmol/L) |  |  |  |  |  |  |  |  |  |  |  |  |  |  |  |  |  |
| SNP rs3737967 ^a^ |  |  |  |  |  |  |  |  |  |  |  |  |  |  |  |  |  |
| CC | 114 | 10.8 ± 1.8 |  | 116 | 12.4 ± 2.6 |  | 41 | 13.6 ± 2.7 |  | 30 | 11.3 ± 2.4 |  | 18 | 12.5 ± 1.9 |  | 27 | 11.8 ± 2.3 |
| CT + TT | 27 | 11.0 ± 2.1 |  | 25 | 11.8 ± 2.2 |  | 6 | 12.0 ± 1.1 |  | 8 | 12.0 ± 2.9 |  | 5 | 12.3 ± 1.3 |  | 6 | 10.8 ± 2.9 |
| SNP rs1801131 ^a^ |  |  |  |  |  |  |  |  |  |  |  |  |  |  |  |  |  |
| AA | 99 | 10.9 ± 1.9 |  | 100 | 12.1 ± 2.5 |  | 36 | 13.2 ± 2.8 |  | 25 | 11.1 ± 2.1 |  | 13 | 12.3 ± 1.4 |  | 26 | 11.4 ± 2.0 |
| AC + CC | 42 | 10.6 ± 1.8 |  | 41 | 12.8 ± 2.6 |  | 11 | 13.8 ± 1.4 |  | 13 | 12.3 ± 3.1 |  | 10 | 12.6 ± 2.2 |  | 7 | 12.5 ± 3.4 |
| SNP rs9651118 |  |  |  |  |  |  |  |  |  |  |  |  |  |  |  |  |  |
| TT | 53 | 10.7 ± 1.8 |  | 44 | 12.1 ± 2.3 |  | 12 | 13.1 ± 2.5 |  | 9 | 11.4 ± 2.1 |  | 12 | 12.1 ± 1.9 |  | 11 | 11.5 ± 2.5 |
| TC | 71 | 10.9 ± 2.0 |  | 78 | 12.5 ± 2.6 |  | 27 | 13.5 ± 2.4 |  | 24 | 11.6 ± 2.8 |  | 8 | 13.1 ± 1.7 |  | 19 | 11.7 ± 2.5 |
| CC | 17 | 10.8 ± 1.6 |  | 19 | 12.1 ± 2.6 |  | 8 | 13.3 ± 3.6 |  | 5 | 10.9 ± 1.7 |  | 3 | 11.8 ± 0.8 |  | 3 | 11.3 ± 0.3 |
| TC + CC | 88 | 10.9 ± 1.9 |  | 97 | 12.4 ± 2.6 |  | 35 | 13.5 ± 2.6 |  | 29 | 11.5 ± 2.6 |  | 11 | 12.8 ± 1.6 |  | 22 | 11.7 ± 2.3 |
| Abbreviation: N, number; ARC, age-related cataract; PSC, posterior subcapsular; SD, standard deviation.  ^a^ For SNPs rs3737967 and rs1801131, the TT and CC carriers were relatively rare in our population, so we only evaluated the associations of these two SNPs with MTHFR activity concentrations and tHcy levels under a dominant model. | | | | | | | | | | | | | | | | | |
